# Supplementary material for: Exploring techno-economic landscapes of abatement options for hard-to-electrify sectors
Source: Nat Commun. 2025 Apr 28;16:3984. doi: 10.1038/s41467-025-59277-1 (PMC12037817; doi:10.1038/s41467-025-59277-1)
Supplement: Supplementary file 1 — Supplementary Information [file 41467_2025_59277_MOESM1_ESM.pdf]

Supplementary information

Exploring techno-economic landscapes of abatement options for hard-to-electrify sectors

Clara Bachorz[1,2]*, Philipp C. Verpoort[1], Gunnar Luderer[1,2], Falko Ueckerdt[1]

[1]Potsdam Institute for Climate Impact Research, Potsdam, Germany

[2]Global Energy Systems Analysis, Technische Universität Berlin, Berlin, Germany

*Corresponding author: clara.bachorz@pik-potsdam.de

Supplementary Items List

Supplementary Notes

Supplementary Note 1: Techno-economic assumptions for synthetic fuel costs
Supplementary Note 2: Cost and emission factors for fossil fuels
Supplementary Note 3: Impact of the technological bottlenecks on abatement costs
Supplementary Note 4: TEA for the aviation sector
Supplementary Note 5: TEA for the maritime sector
Supplementary Note 6: TEA for the primary steel-making sector
Supplementary Note 7: TEA for the cement sector
Supplementary Note 8: TEA for the chemical feedstocks sector
Supplementary Note 9: Low-emission hydrogen: blue and green hydrogen
Supplementary Note 10: Non-fossil CO_2: biogenic and atmospheric

Supplementary Tables

Supplementary Table 1: Techno-economic parameters used for synthetic fuel synthesis
Supplementary Table 2: Cost and emission factors for the fossil fuel considered in this study
Supplementary Table 3: Key parameters used for the aviation sector
Supplementary Table 4: Breakdown of the different cost components for the aviation sector
Supplementary Table 5: Key parameters used for the maritime sector
Supplementary Table 6: Key parameters used for the steel sector
Supplementary Table 7: Key parameters used for the cement sector
Supplementary Table 8: Key parameters used for the chemical feedstock sector
Supplementary Table 9: Key parameters used for calculating blue hydrogen costs
Supplementary Table 10: Cost and availability of various CO_2 biogenic sources in 2050

Supplementary Figures

Supplementary Figure 1: Breakdown of the abatement costs calculation.
Supplementary Figure 2: Sensitivity of the mitigation landscapes to the CO_2 transport and storage cost.
Supplementary Figure 3: Greenfield and brownfield comparison for the steel sector, under the standard case and the climate-neutral case.
Supplementary Figure 4: Sensitivity analysis of the steel sector results depending on the CAPEX of carbon capture on a blast furnace and basic oxygen furnace
Supplementary Figure 5: Greenfield and brownfield comparison for the chemical feedstock sector, under the standard case and the climate-neutral case.
Supplementary Figure 6: Cost of different low-emission hydrogen production pathways.
Supplementary Figure 7: Competition between blue and green hydrogen-based steel.
Supplementary Figure 8: Non-fossil CO_2 supply curve for 2050.
Supplementary Figure 9: Mitigation landscapes with a higher CCU attribution of 85%

Supplementary Note 1. Techno-economic assumptions for synthetic fuel costs

We carried out a techno-economic analysis (TEA) to calculate the levelized cost of liquified low-emission H2, low-emission ammonia, synthetic methanol, and synthetic jet fuel. For the carbonaceous fuels (methanol and jet fuel), the CO_2 used can be sourced from a fossil carbon capture and utilization (fossil CCU) point source or non-fossil sources (from direct-air capture or biogenic sources). Synfuels synthesized using fossil CCU CO_2 are fossil CCU synfuels, while the other pathway produces low-emission synfuels. The techno-economic assumptions used for calculating the levelized costs are based on a literature review, as cited below in Supplementary Table 1.

Supplementary Table 1. **Techno-economic parameters used for synthetic fuel synthesis**

**Process**	**Parameter type**	**Parameter**	**Value**	**Source**	**Comment**
**H2 liquefaction**	Economic	Liquefaction cost (capital expenses)	€37.5 MWh_{H2}^{-1}	Connelly et al.[1]	Value given in the paper: $1.4 kg_{H2}^{-1}
	Technical	Electricity demand	0.35 MWh MWh_{H2}^{-1}	Ghafri et al.[2]	converted from kWh kg_{H2}^{-1} using the LHV. The value given is from the same established plant as Connelly et al.
		Hydrogen demand	1 MWh MWh_{H2}^{-1}		
**Ammonia synthesis (using an air separation unit)**	Economic	CAPEX	€161.0 MWh_{NH3}^{-1}	Grahn et al.[3]	
		OPEX	4%	Grahn et al.[3]	
		Lifetime	25 years	Grahn et al.[3]	
	Technical	Electricity demand	0.089 MWh MWh_{NH3}^{-1}	Stolz et al.[4]	0.063 MWh used for the plant operation, and 0.026 MWh for operating the ASU.
		Hydrogen demand	1.148 MWh_{H2} MWh_{NH3}^{-1}	Stolz et al.[4]	

**Methanol synthesis**	Economic	CAPEX	€79.9 MWh_{CH3OH}^{-1}	Grahn et al.[3]	
		OPEX	4%	Grahn et al.[3]	
		Lifetime	25 years	Grahn et al.[3]	
	Technical	Electricity demand	0.067 MWh MWh_{CH3OH}^{-1}	Stolz et al.[4]	Excess heat produced is considered waste heat.
		Hydrogen demand	1.23 MWh_{H2} MWh_{CH3OH}^{-1}	Stolz et al.[4]	
		CO_2 demand	0.27 tCO_2 MWh_{CH3OH}^{-1}	Stolz et al.[4]	
**Jet fuel synthesis**	Economic	CAPEX	€114.16 MWh_{kero}^{-1}	Grahn et al.[3]	
		OPEX	4%	Grahn et al.[3]	
		Lifetime	25 years	Grahn et al.[3]	
	Technical	Electricity demand	0.109 MWh MWh_{kero}^{-1}	Bube et al.[5]	
		Hydrogen demand	0.092 MWh_{H2} MWh_{kero}^{-1}	Bube et al.[5]	
		Methanol demand	1.277 MWh_{CH3OH} MWh_{kero}^{-1}	Bube et al.[5]	

CAPEX: capital expenditure, OPEX: operational expenditure, NH3: low-emission ammonia, H2: low-emission hydrogen, CH3OH: Methanol, kero: Kerosene.

Supplementary Note 2. Cost and emission factors for fossil fuels

Fossil fuel prices and emission intensity are required for the techno-economic analysis of the hard-to-electrify sectors. This is relevant for the fossil references used (for example heavy fuel oil for maritime transport) and for some abatement options which require fossils (for example natural gas use for carbon capture in a blast furnace). We collected cost and emission data, taking average fossil prices from 2010 to 2020 where possible. All the data used is shown in Supplementary Table 2.

Supplementary Table 2. **Cost and emission factors for the fossil fuel considered in this study**

	**Cost (2010-2020 average, unless indicated otherwise)**	**CO_2 emissions**	**Fuel specification**
**Coking coal**	€148.2 t^{-1} (*)	2.84 tCO_2 t^{-1} (**)	A mix of 60%w hard coking coal and 40%w soft coking coal. Overall carbon content of 78.9%, and heating value of 31.10 MJ kg^{-1}.
**PCI**	€127.6 t^{-1} (*)	3.13 tCO_2 t^{-1} (**)	Coal with a carbon content of 87%, and a heating value of 33.37 MJ kg^{-1}.
**Bituminous coal**	€8.91 MWh^{-1} [6]	0.336 tCO_2 MWh^{-1} [7]	
**Natural gas**	€20.38 MWh^{-1} [8]	0.202 tCO_2 MWh^{-1} [7]	
**Heavy fuel oil**	€52.03 MWh^{-1} [9] (***)	0.279 tCO_2 MWh^{-1} [7]	With low sulphur content, according to the 2020 IMO guidelines
**Naphtha**	€518 t^{-1} [10]	3.29 tCO_2 t^{-1} [7]	
**Jet fuel**	€51.94 MWh^{-1} [11]	0.32 tCO_2 MWh^{-1} [12]	

PCI: pulverized coal injection.
*: cost estimation taken directly from[13], and converted from $\$_{2010}$ to $€_{2020}$ using the conversion rate: $\$_{2010}1$ =$€_{2020}$ 0.86. See the caption of Supplementary Table 4 for more conversion details.
**: The emission factor is calculated from the carbon content of the type of coal used, which is found in [13].
***: Average only taken over 3 years (01/09/2021-01/09/2023), average conversion rate used: $1 = €0.93

Supplementary Note 3: Impact of the technological bottlenecks on abatement costs

In this work, we identify three key bottleneck technologies that have a large cost impact on the abatement options we consider: low-emission hydrogen, non-fossil CO_2 and CO_2 transport and storage cost. The costs of non-fossil CO_2 and low-emission hydrogen account for most of the cost difference between different abatement options, as can be seen in Supplementary Fig. 1. The cost of CO_2 transport and storage is less critical, but can impact the competition between given mitigation options, such as carbon capture and storage and CCU, or synfuels and carbon dioxide removals (CDR) compensation, as shown in Supplementary Fig. 2.

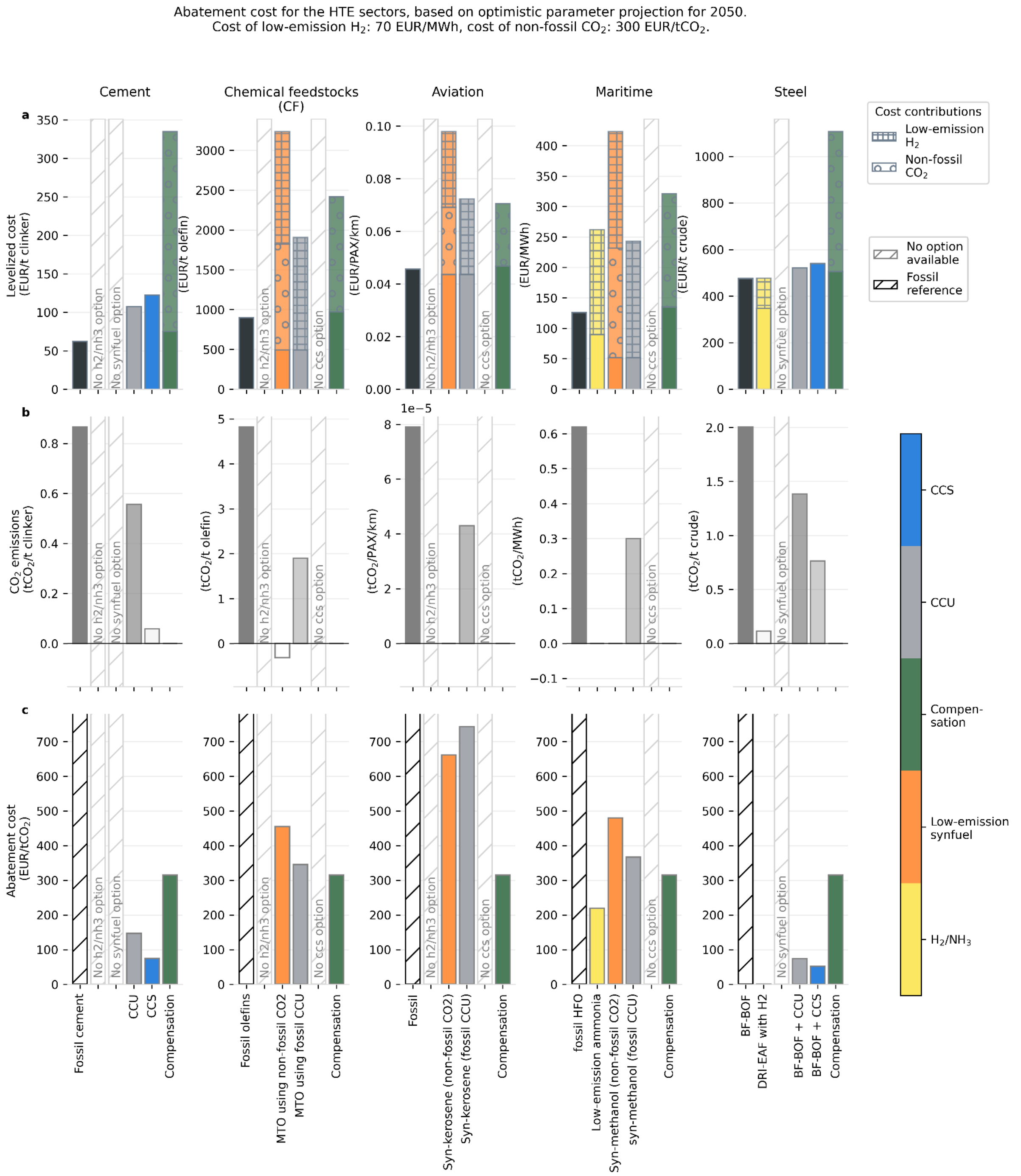

Supplementary Figure 1. **Breakdown of the abatement cost calculation. (a)** shows the levelized cost of the product (LCOX) concerned, with the cost contribution from low-emission hydrogen (H2) and non-fossil CO_2 shown. **(b)** shows the CO_2 emissions, and **(c)** the abatement costs. The light grey shaded diagonally shaded bars indicate where there is no option of a given type available to the sector. The black shaded diagonally bars indicate the fossil reference for the abatement cost calculation, for which the metric is not mathematically defined: we indicate the cost and emissions associated with this option in the top two rows. CCS: carbon capture and storage, CCU: carbon capture and utilization, NH3: ammonia. MTO: methanol-to-olefins, BF-BOF: blast furnace and basic oxygen furnace, DRI-EAF: direct reduction of iron and electric arc furnace.

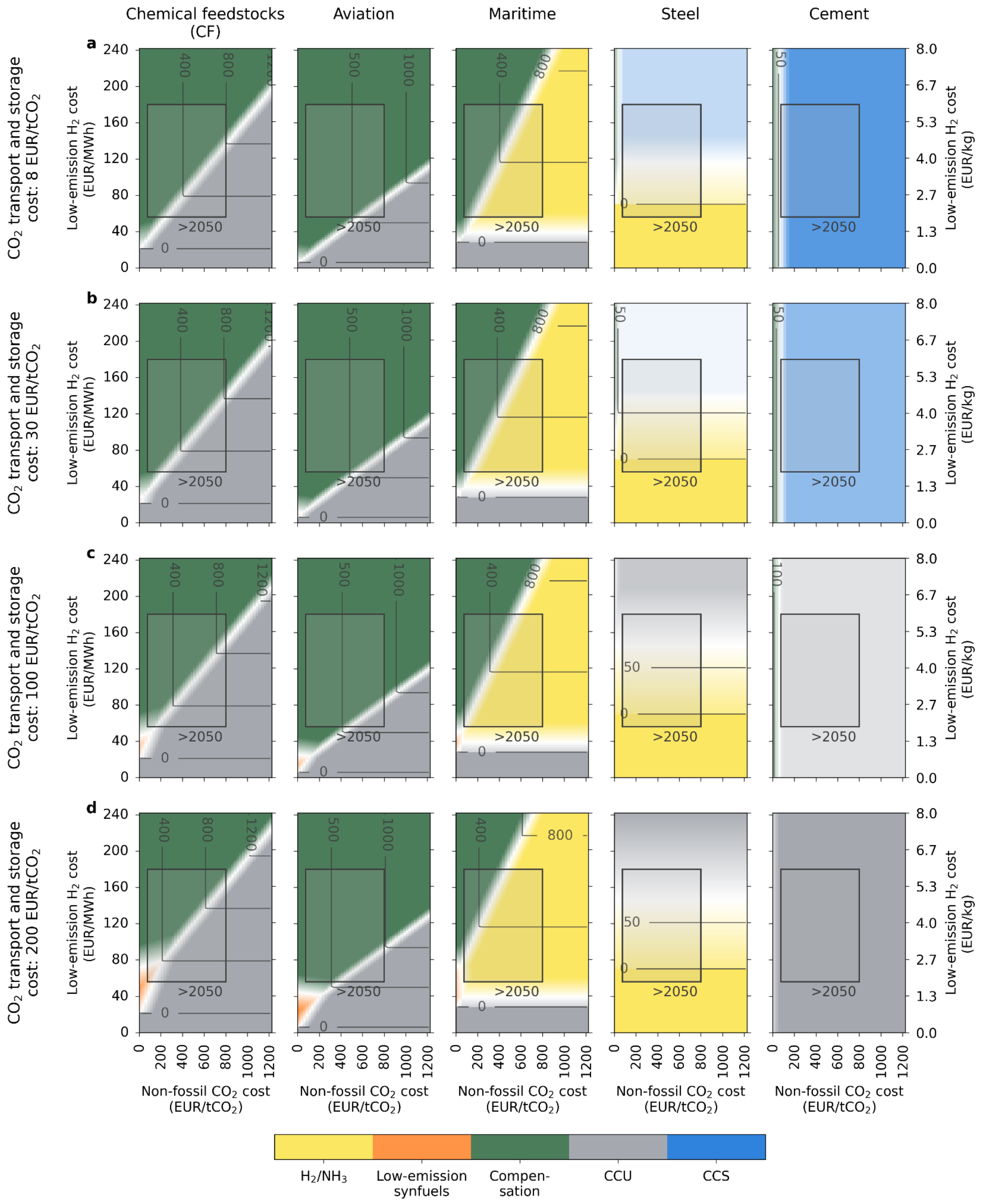

Supplementary Figure 2. **Sensitivity of the mitigation landscapes to the CO_2 transport and storage cost.** Here, the calculation is repeated using a cost of **(a)** €8 tCO_2^{-1}, **(b)** €30 tCO_2^{-1}, **(c)** €100 tCO_2^{-1} and **(d)** €200 tCO_2^{-1}. H2: hydrogen, CCS: carbon capture and storage, CCU: carbon capture and utilization, NH3: ammonia.

Supplementary Note 4. TEA for the aviation sector

We follow the AEA's standard method for evaluating the direct operating costs (DOC) (AEA, "Short-Medium range aircraft – AEA requirements", December 1989), a method which is further detailed by Al-Shamma et al.[14] For the technical and economic parameters, we use the data in Dahal et al.[15] All costs taken are originally in $\$_{2010}$ and are converted to $€_{2020}$ at the end of this Note.

We consider a long-range (LR) aircraft and assume a flying distance corresponding roughly to a Hong Kong to San Francisco flight. For the aviation sector, three pathways are compared to the fossil fuel route: compensation, fossil CCU syn-jet fuel and low-emission syn-jet fuel. Both syn-jet fuels are drop-in fuels, and use the same aircraft type as in the fossil case. Additionally, we calculated the relevant data for a liquified H2 aircraft, but did not use it in the final analysis as this option remains at a low technology readiness level relative to the other options[16]. The key parameters are summarised in Supplementary Table 3.

From the parameters above, we first calculate the utilization (U) according to the AEA's definition, given in Supplementary Equation (1).

$$U = (\text{annual aircraft availability} * \text{block time}) / (\text{block time} + \text{TAT}) \qquad (1)$$

Therefore U = 5317.7 h year^{-1}.

Insurance, Interest, and depreciation:

First, the aircraft cost is defined as:

$$C_{\text{AC}} = 10^6 \times (1.18 \times m_{\text{oe}}^{0.48} - 116), \qquad (2)$$

where m_{oe} is the operating empty weight. The aircraft cost C_{AC} is therefore M$233.5 for synthetic jet fuel and M$267.3 for LH2 aircraft. We then calculate the depreciation, hull insurance, and interest costs using Supplementary Equations (3), (4) and (5).

$$\text{Depreciation} = (0.9 \times \text{bloc time} \times \text{TI}) / (\text{lifespan} \times U) \qquad (3)$$

$$\text{Interest} = (\text{interest rate} \times \text{bloc time} \times \text{TI}) / U \qquad (4)$$

$$\text{Insurance} = (0.005 \times C_{\text{AC}}) / U \qquad (5)$$

Other fees:

First, the cost of the cabin and pilot crew need to be accounted for. This cost is calculated using:

$$\text{Cost cabin crew} = \text{no. cabin crew} \times \text{salary} \times \text{block time} \qquad (6)$$

$$\text{Cost pilot crew} = \text{no. pilot crew} \times \text{salary} \times \text{block time} \qquad (7)$$

The following fees are also considered (Supplementary Equations (8), (9), (10)):

$$\text{Navigation fee} = 0.5 \times \left(\text{study length(km)} \times \sqrt{MTOW(lbs) / 50 \times 1000}\right) \qquad (8)$$

$$\text{Landing fee} = 7.8 \times \text{MTOW(lbs)} / 1000 \quad (9)$$

$$\text{Ground handling fee} = 0.1 \times \text{payload weight} \quad (10)$$

Fees not included in this work: The AEA method also includes the calculation of material and maintenance fees to the complete calculation of the DOC. However, according to Dahal et al., this is a negligible component of the total DOC, and we do not include it in our calculation.

Total costs (excluding fuel costs):

Supplementary Table 4 shows the breakdown of all operating costs. The conventional aircraft, which can be used with both conventional jet fuel or synthetic jet fuel, costs a total of c€3.28 PAX^{-1} km^{-1}, outside fuel costs. The liquified H2 aircraft is a bit more expensive, at €3.54 PAX^{-1} km^{-1}.

The fuel cost component is calculated from the block energy use given in Supplementary Table 3, and from the fuel costs calculated from the parameters highlighted in the previous section.

Supplementary Table 3. **Key parameters used for the aviation sector**

	Parameter name	Unit	LR Conventional jet-fuel / syn-jet fuel aircraft	LR liquified H2 aircraft
**Common parameters**	Segment length	km	11126.8	
	Annual aircraft availability	hour	6500	
	Block time	hour	13.5	
	TAT	hour	3	
	Lifespan	year	14	
	Interest rate	%	5	
	No. passengers	PAX	325	
	No. pilot crew	N/A	2	
	No. cabin crew	N/A	9	
	Lifespan	year	14	
**Aircraft-specific parameters**	Operating empty weight	kg	141 000	170 921
	Payload weight	kg	30 875	30 875
	MTOW	kg	280 000	243 500
	Block energy use	$MJ\ PAX^{-1}\ km^{-1}$	0.885	0.947
**Cost parameters**	Pilot crew salary	$\$_{2010}\ hour^{-1}$	200	
	Cabin crew salary	$\$_{2010}\ hour^{-1}$	60	
	Total investment costs (TI)	$M\$_{2010}$	335.6	379.92

All assumptions are taken from Dahal et al. LR: long-range, H2: hydrogen, PAX: person, TAT: turnaround time, MTOW: maximum take-off weight.

Supplementary Table 4. **Breakdown of the different cost components for the aviation sector**

		**Conventional/syn-jet fuel aircraft**	**Liquid hydrogen aircraft**
**Financial cost**	Depreciation	$\$_{2010}$54770 / c€$_{2020}$1.303 PAX^{-1} km^{-1}	$\$_{2010}$62003.6 / c€$_{2020}$1.475 PAX^{-1} km^{-1}
	Interest	$\$_{2010}$42599 / c€$_{2020}$1.013 PAX^{-1} km^{-1}	$\$_{2010}$48222 / c€$_{2020}$1.147 PAX^{-1} km^{-1}
	Insurance	$\$_{2010}$219.5 / c€$_{2020}$0.005 PAX^{-1} km^{-1}	$\$_{2010}$251.3 / c€$_{2020}$0.006 PAX^{-1} km^{-1}
**Fees & charges**	Crew cost	$\$_{2010}$12690 / c€$_{2020}$0.302 PAX^{-1} km^{-1}	$\$_{2010}$12690 / c€$_{2020}$0.302 PAX^{-1} km^{-1}
	Navigation fee	$\$_{2010}$19545.2 / c€$_{2020}$0.465 PAX^{-1} km^{-1}	$\$_{2010}$18226.8 / c€$_{2020}$0.433 PAX^{-1} km^{-1}
	Landing fee	$\$_{2010}$4813.5 / c€$_{2020}$0.114 PAX^{-1} km^{-1}	$\$_{2010}$4186.1 / c€$_{2020}$0.1 PAX^{-1} km^{-1}
	Ground handling fee	$\$_{2010}$3087.5 / c€$_{2020}$0.073 PAX^{-1} km^{-1}	$\$_{2010}$3087.5 / c€$_{2020}$0.073 PAX^{-1} km^{-1}
**Total**		$\$_{2010}$137725 / c€$_{2020}$3.275 PAX^{-1} km^{-1}	$\$_{2010}$148668 / c€$_{2020}$3.536 PAX^{-1} km^{-1}

The costs are given per flight, in $\$_{2010}$ (units used by Dahal et al.) / c€$_{2020}$ PAX^{-1} km^{-1} (units used in this work). The conversion from $\$_{2010}$ to €$_{2020}$ is done following these exchange rates: Average exchange rate \$ - € in 2010: \$1 = €0.7551. Accounting for inflation, €$_{2010}$1 = €$_{2020}$1.139. Therefore $\$_{2010}$1 =€$_{2020}$ 0.86. c: cent, PAX: person.

Supplementary Note 5. TEA for the maritime transport

Our analysis follows the method and assumptions developed by Korberg et al[17]. The ship modelled in this techno-economic analysis is a two-stroke engine, used for deep-sea shipping. These typically require higher investment costs than four-stroke systems and undergo longer trips. Ferries are not considered in this work, as improvements in electric batteries have meant that ferries are increasingly considered as directly electrifiable[18].

Alternative options not considered:

In this focus study on container ships, we do not consider liquified H2 as a viable option. This is due to the much larger storage size required for this fuel, as a result of its lower volumetric energy density[19], and the challenge of developing appropriate bunkering infrastructure[20]. We instead consider low-emission ammonia from low-emission H2 as a non-carbonaceous option, which has been shown by previous literature to be one of the most competitive abatement options[4,17,21]. However, we acknowledge the environmental concerns associated with this option[22,23]. Other studies explore the challenges and opportunities of using LH2 for the maritime sector[20].

Similarly, we do not consider fuel cell (FC) alternatives in this work and focus on internal combustion engine (ICE) options. ICE HFO and diesel engines can be retrofitted to use ammonia and methanol[24] (new methanol retrofit projects have been announced by MAERSK and COSCO[25,26]) as opposed to FC options, which will require almost entirely new systems. However, in the long term, FC engines running on H2 and NH3 will be increasingly attractive, once costs have reduced[27]. They offer a higher fuel efficiency and virtually no emissions.

Summary of assumptions

As a summary, we consider the following abatement options in this work: ICE with synthetic methanol (CO_2 from fossil CCU or non-fossil CO_2 based), ICE with ammonia, and compensation. The fossil reference is an ICE using heavy fuel oil (HFO). Supplementary Table 5 summarises the key TEA assumptions, assuming a ship utilization of 6000 hours year^{-1}.

Supplementary Table 5. **Key parameters used for the maritime sector**

	**ICE HFO**	**ICE Methanol**	**ICE Ammonia**
**CAPEX (engine)**	€460 kW^{-1}	€505 kW^{-1}	€920 kW^{-1}*
**CAPEX (storage)**	€315 kW^{-1}	€540 kW^{-1}	€1035 kW^{-1}
**OPEX**	2.5%	2.5%	4.5%
**Lifetime**	30 years	30 years	30 years
**WACC**	7%	9%	12%
**Fuel demand**	2.22 MWh MWh^{-1}	2.22 MWh MWh^{-1}	2.22 MWh MWh^{-1}

*Own assumption, we include the capital cost of installing an ammonia cracker on board for the ammonia vessel. Indeed, ammonia requires a pilot fuel to combust, which we take to be low-emission H2 cracked from low-emission ammonia onboard. Our estimation is based on previous calculations by De Vries[28]. All other assumptions are taken from Korberg et al. ICE: internal combustion engine, HFO: heavy fuel oil, CAPEX: capital expenditure, OPEX: operational expenditure, WACC: weighted average cost of capital.

Supplementary Note 6. TEA for the primary steel-making sector

In this work, four abatement options for primary steel-making are considered. The first two options are a blast furnace with a basic oxygen oven and a carbon capture plant, where the carbon captured is either transported and stored underground (BF-BOF-CCS), or where the CO_2 is given to another subsector (aviation, maritime, chemical feedstocks) to make new carbon-based products (BF-BOF-CCU). The third option is a direct reduction of iron plant with an electric arc furnace, where the reduction uses low-emission H2 (DRI-EAF-H2). Finally, the last abatement option is compensation. All plants are compared to a standard BF-BOF fossil steel plant.

The data used for the BF-BOF plants (with and without CCS) is based on an average between the IEAGHG Iron and Steel study (EOP-L2 for the CCS/U case)[13] and Fischedick et al[29]. For the H2-DRI-EAF data, an average of three sources was considered: Agora[30], Vogl et al[31]., Fischedick et al[29]., and Jacobash et al[32]. The original data choice and the averaging have been carried out using the open-source techno-economic framework POSTED (https://github.com/PhilippVerpoort/posted). Supplementary Table 6 summarises the final assumptions used.

While we assume greenfield investment for all steel plants in the first part of the analysis, exploring the retrofit case is particularly relevant for the steel sector. In this case, the fossil BF-BOF plant is

fully depreciated, and can be retrofitted with CCS at a lower abatement cost (see Figure 6 in the main text). For the mitigation landscape, where we map out the full range of low-emission H_2 and non-fossil CO_2 costs, we find that CCS becomes more cost-competitive relative to H_2-based steel when no additional conditions are applied (Supplementary Fig. 3a–b). This cost advantage reduces when climate neutrality is required (Supplementary Fig. 3c–d).

Another key parameter which affects the competition between CCS steel and H_2 steel is the CAPEX of BF-BOF-CCS. This parameter is difficult to estimate, as there are currently few existing BF-BOF-CCS plants. To assess how this impacts our results, we vary this parameter by ±50% and find no significant changes in our results (Supplementary Fig. 4).

Supplementary Table 6. **Key parameters used for the steel sector**

	**BF-BOF**	**BF-BOF-CCS(U)**	**DRI-EAF-H2**
**CAPEX**	€684.4 t_{HRC}^{-1} (€$_{2010}$605.7 t_{HRC}^{-1})	€880.6 t_{HRC}^{-1} (€$_{2010}$779.2 t_{HRC}^{-1})	€556 t_{HRC}^{-1}
**OPEX relative**	4.1%	3.8%	3%
**other OPEX**	€70.9 t_{HRC}^{-1} (€$_{2010}$62.7 t_{HRC}^{-1})	€78.1 t_{HRC}^{-1} (€$_{2010}$69.1 t_{HRC}^{-1})	€50.7 t_{HRC}^{-1}
**WACC**	10%	10%	10%
**Lifetime**	25 years	25 years	18 years
**Electricity demand**	/	/	0.68 MWh t_{HRC}^{-1}
**H2 demand**	/	/	1.85 MWh t_{HRC}^{-1}
**Coking coal demand**	0.524 t t_{HRC}^{-1}	0.464 t t_{HRC}^{-1}	/
**PCI coal demand**	0.151 t t_{HRC}^{-1}	0.151 t t_{HRC}^{-1}	/
**Fossil gas demand**	0.21 MWh t_{HRC}^{-1}	1.06 MWh t_{HRC}^{-1}	0.57 MWh t_{HRC}^{-1}
**Iron ore demand**	1.58 t t_{HRC}^{-1}	1.58 t t_{HRC}^{-1}	1.41 t t_{HRC}^{-1}
**Scrap /ferroalloys demand**	0.172 t t_{HRC}^{-1}	0.172 t t_{HRC}^{-1}	0.13 t t_{HRC}^{-1}
**CO_2 captured**	/	1.24 tCO_2 t_{HRC}^{-1}**	/

BF-BOF: blast furnace with a basic oxygen furnace, BF-BOF-CCS(U): BF-BOF with carbon capture and storage (CCS) or utilization (CCU), DRI-EAF-H2: direct reduction of iron with low-emission hydrogen (H2) with an electric arc furnace, CAPEX: capital expenditure, OPEX: operational expenditure, WACC: weighted average cost of capital, PCI: pulverized coal injection, HRC: hot rolled coil. Conversion used: $\$_{2010}1$ = $€_{2010}0.755$. $€_{2010}1$ = $€_{2020}1.139$.
*OPEX includes costs for fixed O&M (maintenance, labour).
** Total emissions from this pathway are 2.07 tCO_2 t_{HRC}^{-1}, so the CCS plant has a 65% capture rate.

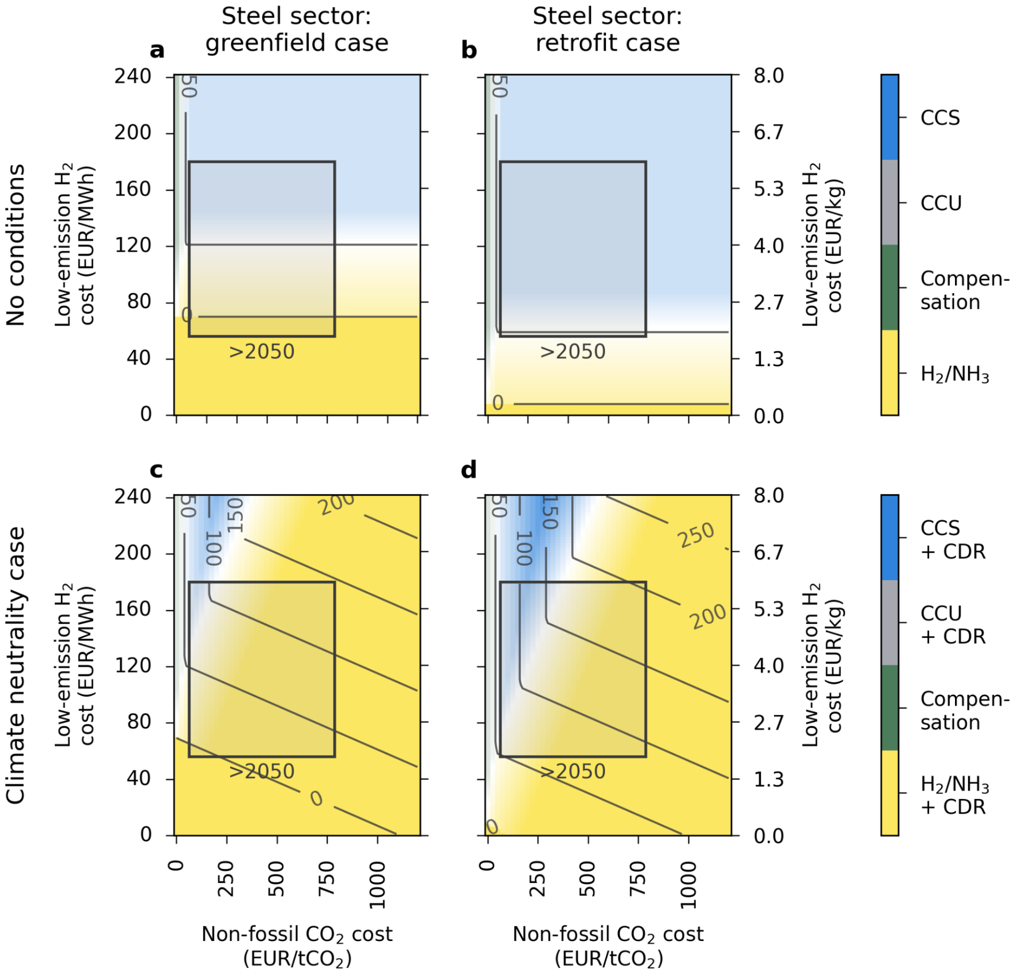

Supplementary Figure 3. **Greenfield and brownfield comparison for the steel sector, under the standard case and the climate-neutral case. (a)** and **(b)** are the standard case. **(c)** and **(d)** are the climate-neutral case, compatibility with climate neutrality is required by compensating for any residual CO_2 emissions using direct-air capture and CO_2 transport and storage (DACCS) or carbon capture and storage (CCS) of biogenic CO_2. CCU: carbon capture and utilization, H2: hydrogen, NH3: ammonia, CDR: carbon dioxide removal (use of DACCS/CCS of biogenic CO_2 for compensating residual emissions).

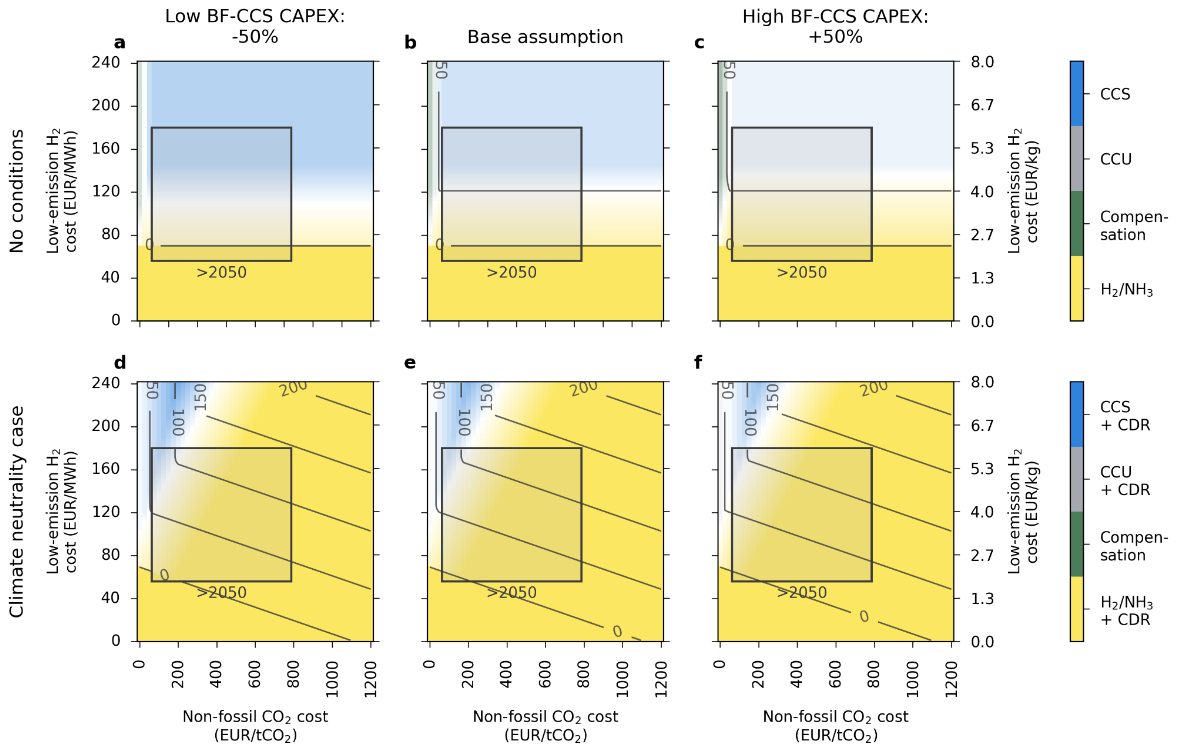

Supplementary Figure 4. **Sensitivity analysis of the steel sector results depending on the CAPEX of carbon capture on a blast furnace and basic oxygen furnace. (a)** and **(d)** show the low-cost assumption (-50% relative to the base case) for the standard and climate-neutral case, respectively. **(b)** and **(e)** show the base assumption, while **(c)** and **(f)** show the high-cost assumption (+50% relative to the base cost). In the climate-neutral case, compatibility with climate neutrality is required by compensating for any residual CO_2 emissions using DACCS or carbon capture and storage (CCS) of biogenic CO_2. DACCS: direct-air capture and CO_2 transport and storage, CCU: carbon capture and utilization, H2: hydrogen, NH3: ammonia, CDR: carbon dioxide removal (compensation of residual emissions using DACCS or CCS of biogenic CO_2).

Supplementary Note 7. TEA for the cement sector

For cement production, three alternative production routes are considered: CCS, CCU, and compensation. The fossil reference is a standard cement kiln. All techno-economic data used, for the standard and CCS(U) plant, follows De Lena et al.,[33] who compare a standard cement plant against 3 different installations of CCS using calcium looping (CaL) processes. We only consider the integrated CaL plant, for two reasons: it offers the largest emissions reduction potential (93%), even more so when considering an electricity mix with a low CO_2 intensity. On the other hand, integrated CaL may not be the easiest CCS option for retrofitting current fossil plants[34]. More technical details on alternative carbon capture methods in cement production are available in Voldsund et al.[34] and Gardarsdottir et al.[35] The techno-economic assumptions taken are summarized in Supplementary Table 7.

Supplementary Table 7. **Key parameters used for the cement sector**

	**Standard cement plant**	**Integrated CaL plant (CCS/CCU)**
**CAPEX**	€210.47 $t_{clinker}^{-1}$	€437.69 $t_{clinker}^{-1}$
**OPEX (excl. energy and electricity costs)**	€27.0 $t_{clinker}^{-1}$	€41.4 $t_{clinker}^{-1}$
**Lifetime**	25 years	25 years
**WACC**	8%	8%
**Electricity demand**	0.131 MWh $t_{clinker}^{-1}$	0.174 MWh $t_{clinker}^{-1}$
**Bituminous coal demand**	0.9 MWh $t_{clinker}^{-1}$	1.51 MWh $t_{clinker}^{-1}$
**Direct CO_2 emissions (calcination only)**	0.563 tCO_2 $t_{clinker}^{-1}$	0.548 tCO_2 $t_{clinker}^{-1}$
**CO_2 captured**	/	0.997 tCO_2 $t_{clinker}^{-1}$

CaL: calcium looping, CCS: carbon capture and storage, CCU: carbon capture and utilization, CAPEX: capital expenditure, OPEX: operational expenditure, WACC: weighted average cost of capital. Values were converted from $€_{2014}$ to $€_{2020}$ using an exchange rate of $€_{2014}$ 1 = $€_{2020}$1.0624.

Supplementary Note 8. TEA for the chemical feedstocks sector

This work focuses on the production of higher-value chemicals (HVCs), and more specifically olefin production. Indeed, other products from the chemical sector such as methanol, ammonia and chlorine, are simpler. They consequently require fewer synthesis steps for both the fossil and green alternative pathways. Ammonia and chlorine also do not contain any carbon, thereby removing the need for a biogenic or atmospheric carbon source. Additionally, in terms of the usage of fossil chemical feedstocks, HVCs constitute the bulk of the demand, taking up 62% of the global demand for the chemical sector[36].

HVC production results in three types of CO_2 emissions: first, energy-related emissions, from the use of fossil fuels in heat or steam generation. Then, process emissions reflect the difference in carbon content between the feedstock and the end-product. And finally, end-of-life emissions, which are either positive if the product is incinerated, or negative if the product is recycled (as the carbon remains stored in the material). Energy and process emissions are usually accounted for and attributed to the HVC production process, but in this work, we additionally consider the emissions from the end-of-life of plastic products.

Focusing on using alternative chemical feedstocks (so-called defossilization), works such as by Kätelhön et al.[37] and Lopez et al.[38] both illustrate that methanol is the key intermediate from which almost all chemical products can be made (to the exception of chlorine and ammonia, which have different low-carbon pathways). Methanol can be used to produce both olefins, through the methanol-

to-olefin (MTO) process, and BTX aromatics through the methanol-to-aromatics (MTA) process. For this analysis, we focus exclusively on olefin production using MTO, for both ethylene and propylene. This is mainly due to the higher technological maturity of the MTO route, which allows for a more reliable analysis compared to MTA, which is still at TRL 7[16].

Therefore, we compare the fossil olefin synthesis route of naphtha cracking with three alternative options. The first two are the use of synthetic methanol in the MTO process, using methanol produced either from fossil CO_2 CCU sources (fossil CO_2 CCU syn-methanol), or from non-fossil CO_2 (low-emission syn-methanol). The final option is emissions compensation. Key techno-economic assumptions for each option are detailed below in Supplementary Table 8. The TEA for the fossil naphtha cracking route is based on Spallina et al.[39] For the MTO route, we follow Dutta et al.[40]

The method used for accounting emissions is the following:

Energy emissions:
Electricity demand is accounted for in both cases. Naphtha cracking is, outside of electricity, a self-sustaining process, with 95% of the energy required provided by the combustion of fuel gases[41]. For the MTO process, some low-pressure steam is required in the process[40]. But as such a process is directly electrifiable[42], the provision of steam for the MTO process is not taken into account.

Process emissions:
Process emissions are calculated from the difference in carbon content between the feedstock used (naphtha or methanol) and the final ton of olefin, which as explained below is assumed to contain 3.14 $tCO_2\ t_{olefin}^{-1}$. The process emissions are therefore 2 $tCO_2\ t_{olefin}^{-1}$ for the naphtha cracking route, and 0–1.29 $tCO_2\ t_{olefin}^{-1}$ for the MTO route (depending on the emission intensity of methanol, with pure fossil methanol being the higher end of the range).

End-of-life emissions:
The end-of-life emissions arising from the incineration or recycling of the products are calculated based on the emission factor of a ton of olefins. This amount is calculated to be 3.14 $tCO_2\ t_{olefin}^{-1}$, based on the carbon content of an ethylene molecule. Of this amount, which would occur as a result of full combustion, we assume that 10% of the CO_2 is recycled, leading to total end-of-life emissions of 2.83 $tCO_2\ t_{olefin}^{-1}$. This assumption arises from the fact that as of 2014, roughly 60% of plastics were discarded, the remainder split between recycling and incineration[42]. Additionally, as current recycling practices suffer from the mixing of polymer types, leading to secondary plastics of lower quality[42], a conservative assumption of a 10% effective recycling rate was used.
The 2.83 $tCO_2\ t_{olefin}^{-1}$ emitted is only in the case of a full fossil-based feedstock (fossil naphtha or fossil methanol). In the case of low-emission synthetic methanol for example, the net CO_2 emissions would be -0.31 $tCO_2\ t_{olefin}^{-1}$, as a small portion of CO_2 from the atmosphere is "stored" in the 10% fraction of recycled plastics. In the case of syn-methanol made using fossil CO_2, the emissions depend on the CCU attribution fraction.

Similarly to the steel sector, evaluating the impact of retrofits is important. If an existing naphtha cracking plant is available, switching to a new MTO plant incurs additional capital expenses. However, we find that this has little impact in the final abatement cost comparison (Supplementary Fig. 5). In the chemical sector, feedstocks are the largest cost component, with capital expenditure being significantly smaller.

Supplementary Table 8. **Key parameters used for the chemical feedstock sector**

	**Naphtha cracking route**	**MTO route**
**CAPEX**	€514.2 t_{olefin}^{-1}	€662.5 t_{olefin}^{-1}
**OPEX**	4.5%	/
**Other OPEX***	/	125.7 t_{olefin}^{-1}
**Lifetime**	25 years	20 years
**WACC**	8%	8%
**Electricity demand**	0.28 MWh t_{olefin}^{-1}	0.72 MWh t_{olefin}^{-1}
**Naphtha demand**	1.56 t t_{olefin}^{-1}	/
**Methanol demand**	/	16.41 MWh t_{olefin}^{-1}

MTO: Methanol to olefin, CAPEX: capital expenditure, OPEX: operational expenditure, WACC: weighted average cost of capital. For the naphtha cracking route, all costs are shown here in $€_{2020}$ but are in $€_{2017}$ in the paper. The assumed conversion rate is $€_{2017}1 = €_{2020}1.042$. For the MTO route, the costs are also shown in $€_{2020}$, converted from $\$_{2016}$ using a conversion rate of $\$_{2016}1= €_{2020}0.9582$.
*Other OPEX includes steam, cooling water, and O&M costs.

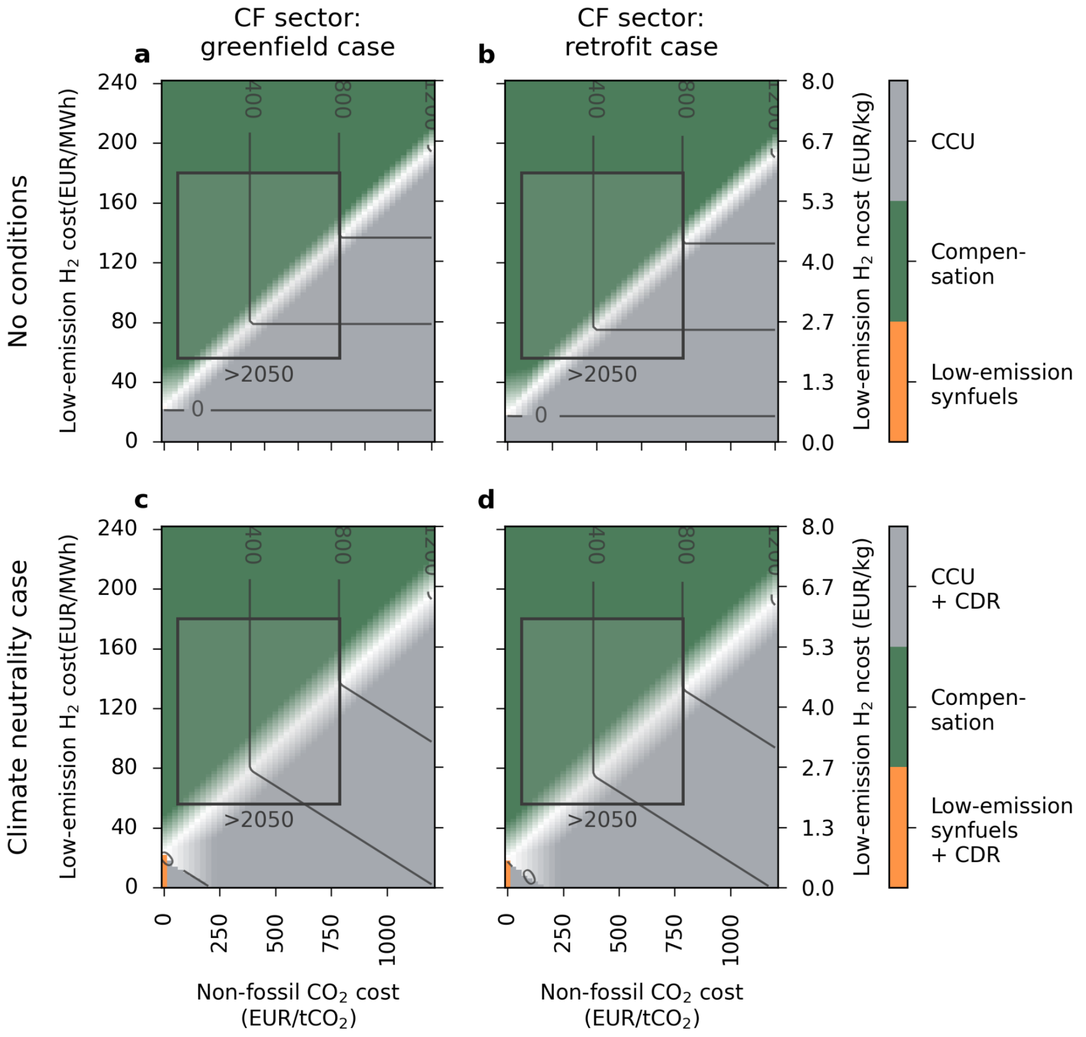

Supplementary Figure 5. **Greenfield and brownfield comparison for the chemical feedstock sector, under the standard case and the climate-neutral case. (a)** and **(b)** show the case with no conditions, for the greenfield and retrofit cases, respectively. **(c)** and **(d)** show the climate neutrality case, for greenfield and retrofit cases, respectively. In the climate-neutral case, compatibility with climate neutrality is required by compensating for any residual CO_2 emissions using direct-air capture and CO_2 transport and storage (DACCS) or carbon capture and storage (CCS) of biogenic CO_2. CCU: carbon capture and utilization, CDR: carbon dioxide removal (use of DACCS/CCS of biogenic CO_2 for compensating residual emissions).

Supplementary Note 9. Low-emission hydrogen: blue and green hydrogen

The two major production routes of low-emission hydrogen considered in this work are green and blue hydrogen. Specifically, green hydrogen is produced using electrolysis with renewable electricity. Blue hydrogen uses steam methane reforming (SMR) in combination with carbon capture and storage (CCS) at high capture rates (>90%). The CO_2 captured is then transported and stored.

<u>Blue hydrogen competitiveness</u>

Whilst blue hydrogen has often been considered a promising option, particularly as a cheaper alternative to green hydrogen[43], it has so far failed to materialize on the market. For example, the IEA Hydrogen Project Database has 198 low-emission H2 projects registered as operating or having reached a final investment decision. Of those, 157 projects were electrolysis-based, whereas 19 projects, or less than 10%, were announced as using natural gas with CCUS. Additionally, other works have highlighted the limitations of blue H2 due to fossil lock-in risks or methane leakage, which dramatically worsens blue H2's climate footprint[44,45].

Here, we offer a short analysis of blue hydrogen cost-competitiveness and the prerequisites for it to be a true competitor to green H2. We estimate the cost of blue hydrogen using parameters corresponding to a high CO_2 capture rate plant (>90%). We use an average between the IEAGHG and Lewis et al.[46,47] The original data choice and the averaging have been carried out using the open-source techno-economic framework POSTED (https://github.com/PhilippVerpoort/posted). Supplementary Table 9 summarises the final assumptions used. Using our basic assumptions, a fossil gas price of €20.4 MWh^{-1} and a CO_2 transport and storage cost of €15 tCO_2^{-1}, result in a blue hydrogen cost of €57 MWh_{H2}^{-1}.

We additionally conduct a more complete cost analysis, with results shown in Supplementary Fig. 6. For the cost of blue hydrogen (blue boxes), the cost of natural gas varies between €10 – €40 MWh^{-1}. Additionally, we show the impact of adding CO_2 pricing of methane leakage emissions, using the global warming potential of methane for 100 years (GWP 100), and assuming a pessimistic CO_2 pricing scenario of €200 tCO_2^{-1}. Based on these assumptions, we see that blue hydrogen becomes less cost-competitive if methane leakage rates are not minimized (kept well under 3%), and reaches a mean cost of €80 MWh^{-1}. This cost analysis also takes relatively optimistic assumptions: it has been argued that the GWP20 may be a better metric to estimate blue hydrogen's climate impact[44], which would further reduce blue hydrogen's cost competitiveness.

Blue hydrogen and DRI-EAF steel

Blue hydrogen with DRI-EAF could be an interesting abatement option for primary steel production. If green hydrogen remains expensive in the next decades, low-emission blue hydrogen (>90% capture rate) could serve as a short-term replacement. This would still abate a large portion of CO_2 emissions from steel production, and additionally motivate investments in new DRI-EAF plants by providing an alternative to green hydrogen, should it fail to materialize at scale.

However, methane leakage emissions in the blue hydrogen supply chain compromise its cost competitiveness relative to green hydrogen. We explore how this affects the cost-effectiveness of DRI-EAF with blue or green hydrogen by varying the methane leakage rate between 0%, 0.1%, and 3%. We calculate the mitigation landscape across three cases: base case, climate neutrality using the GWP100 of methane, and climate neutrality using the GWP20 (Supplementary Fig. 7).

In the base case, we see that DRI-EAF with blue hydrogen dominates the mitigation landscape, replacing BF-BOF-CCS from the mitigation landscape (Supplementary Fig. 7a–c). Previously, BF-BOF-CCS was the most cost-effective abatement option for low-emission hydrogen costs above €120 MWh^{-1} (see main text). DRI-EAF with green hydrogen becomes the most cost-effective abatement option at a green hydrogen cost of €57 MWh^{-1}.

However, in the case of a supply chain with high methane leakage, blue hydrogen loses its cost advantage over green hydrogen in steel when requiring climate neutrality and compensating for these

methane leakage emissions. For low methane leakages of 0.1%, requiring climate neutrality does not impact the cost-competitiveness of blue hydrogen DRI-EAF significantly, and it remains the dominant abatement option (Supplementary Fig. 7d–e and g–h). However, in the case of high methane leakage rates of 3%, blue hydrogen is strongly impacted, both when using the GWP100 of methane (Supplementary Fig. 7f) and the GWP 20 (Supplementary Fig. 7i). In the GWP20 case, green hydrogen DRI-EAF is the most cost-efficient abatement option for high green hydrogen costs of €5 kg^{-1}, illustrating how blue hydrogen's cost competitiveness suffers when methane leakage rates are high. Overall, depending on what CDR compensation cost may be achievable, and what methane leakage rate is tolerated across the blue hydrogen value chain, we see that green hydrogen DRI-EAF could still be the most cost-effective option for greening steelmaking.

Supplementary Table 9. **Key parameters used for calculating blue hydrogen costs**

	**Steam methane reforming with carbon capture**
**CAPEX**	€164.9 MWh_{H2}^{-1}
**OPEX**	€7.2 MWh_{H2}^{-1}
**Lifetime**	27.5 years
**WACC**	7%
**Capacity factor**	92.5%
**Fossil gas demand**	1.51 MWh MWh_{H2}^{-1}
**CO_2 capture rate**	93.25%

CAPEX: capital expenditure, OPEX: operational expenditure, WACC: weighted average cost of capital.

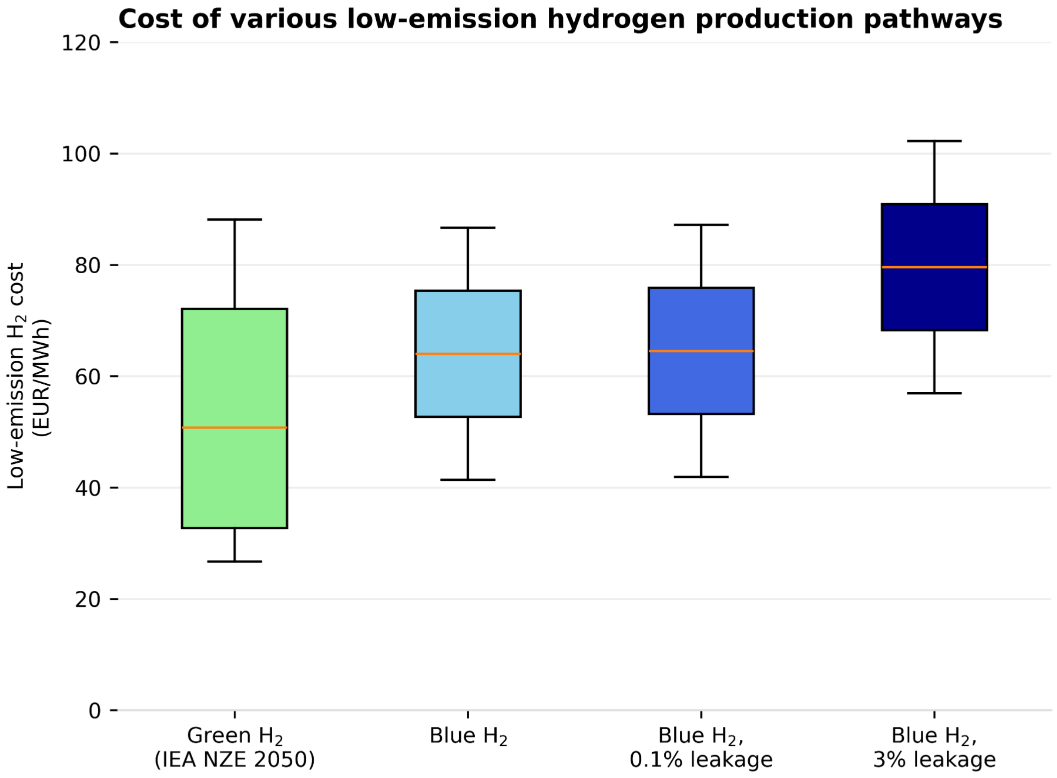

Supplementary Figure 6. **Cost of different low-emission hydrogen production pathways.** The cost of green hydrogen shown is from the IEA's Net zero emission 2050 scenario. Blue hydrogen costs are calculated using the assumptions detailed in the corresponding section in the Supplementary Information, with an added CO_2 cost based on the global warming potential of 100 years of methane, under different methane leakage rates. CO_2 pricing is assumed to be at €200 tCO_2^{-1}. For blue hydrogen, we assume gas costs between €10 MWh^{-1} and €40 MWh^{-1} to assess the uncertainty around the total cost. H2: hydrogen.

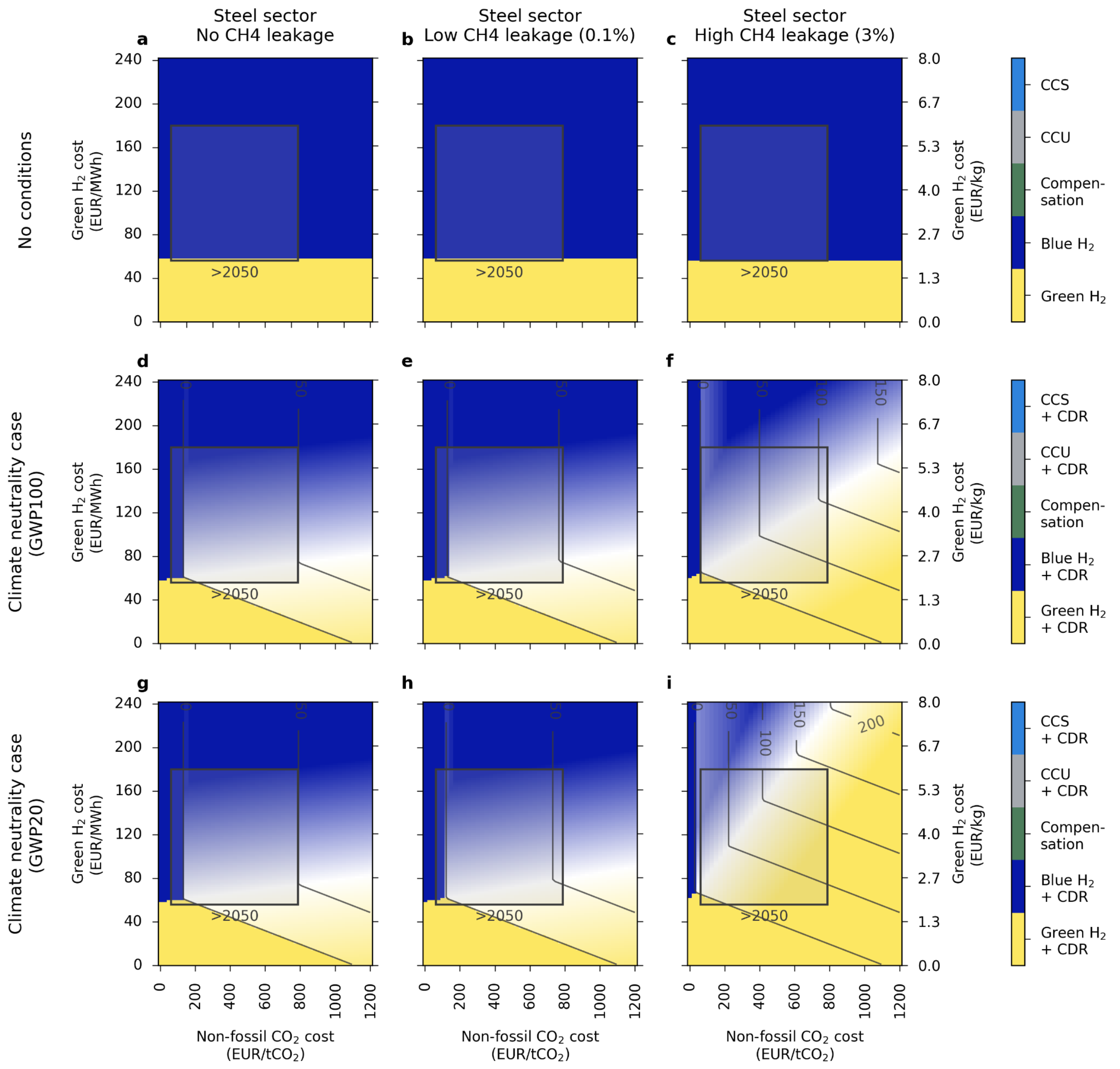

Supplementary Figure 7. **Competition between blue and green hydrogen-based steel.** The methane (CH4) leakage rate associated with blue hydrogen production is differentiated in three cases (horizontally). **(a) (d)** and **(g)** for no methane leakage. **(b) (e)** and **(h)** for low methane leakage of 0.1%. **(c) (f)** and **(i)** for high methane leakage of 3%. In addition, three mitigation cases are represented: **(a-c)** show the standard case of potentially incomplete emission reduction where residual emissions are neither priced nor compensated. **(d-f)** show the climate neutrality case (full emission reduction), using the global warming potential of methane over 100 years (GWP100) for the calculation of residual emissions, which are then compensated using carbon dioxide removal (CDR). **(g-i)** show a climate neutrality case (full emission reduction), using the global warming potential of methane over 20 years (GWP20) for the calculation of residual emissions, which are then compensated using carbon dioxide removal (CDR). H2: hydrogen, CCS: carbon capture and storage, CCU: carbon capture and utilization.

Supplementary Note 10. Non-fossil CO_2: biogenic and atmospheric

Non-fossil CO_2 is a crucial resource for mitigating the hard-to-electrify sectors, as it is required for two mitigation options: emission compensation and low-emission synfuels / synthetic chemicals. For compensation, which is an option available to all sectors, the CO_2 is stored underground. For low-emission synfuels and syn-chemicals, CO_2 is used to make either syn-methanol for the maritime or chemical sector, or syn-jet fuel for the aviation sector.

However, identifying both a cost-effective and widely available source of non-fossil CO_2 is a complicated task. There are two main sources typically considered: biogenic CO_2 or atmospheric CO_2. Biogenic CO_2 refers to the capture of CO_2 released by processing biomass: the largest sources being the production of bio-ethanol, biogas, pulp and paper, and waste-to-energy plants. Electricity and heat production from biomass, with subsequent carbon capture, is an additional source. Atmospheric CO_2, on the other hand, is captured from the atmosphere using direct-air capture (DAC), a technology still in its infancy.

While biogenic CO_2 is more cost-effective than atmospheric CO_2, its availability is intrinsically limited. To investigate the amount of biogenic CO_2 available, and its cost, we conducted a short literature review, with the results shown in Supplementary Table 10. We build a supply cost curve for non-fossil CO_2 based on this data, which also indicates the corresponding CO_2 demand in 2050 for low-emission synfuels in transport and syn-chemicals, based on Galimova et al.[48] Based on this cost curve, we see that biogenic CO_2 supply is not sufficient to fulfil non-fossil CO_2 demand in 2050 for the hard-to-electrify sectors, therefore requiring atmospheric CO_2 (Supplementary Fig. 8). We also note that non-fossil CO_2 will also be required for CDR compensation of non-CO_2 emissions, which may use up a significant portion of the available biogenic CO_2.

Furthermore, we note that the amounts of biogenic CO_2 considered here are optimistic. Fuss et al. identify a large uncertainty on total biogenic CO_2 supply, which could be between 0.5 – 5$GtCO_2$ yr^{-1} [49], as opposed to the 4.2$GtCO_2$ yr^{-1} derived from our literature review. Additionally, a report on net-zero maritime transport by Maersk has found a biogenic CO_2 potential for low-emission synfuels of 0.32-0.37$GtCO_2$[50], an order of magnitude below our estimation. A reason for this variability could be the sustainability concerns around biomass usage[51] — for example, a report from the European Federation for Transport and Environment looks at the theoretical and sustainable biogenic CO_2 supply in Europe. They find that sustainability concerns limit the theoretical potential of more than 0.9 $GtCO_2$ yr^{-1} down to 0.13 $GtCO_2$ yr^{-1} [52].

In light of the above, we can conclude that the quantity of biogenic CO_2 available globally is highly uncertain, and even with the most optimistic assumptions, insufficient for the production of syn-chemicals and fuels by 2050. Therefore, once biomass options have realized their full sustainable potential, the marginal abatement cost of the HTE sectors concerned (maritime, aviation, chemical feedstocks) will be set by the cost of low-emission synfuels synthesized using direct-air capture.

An alternative to non-fossil CO_2 would be to use CO_2 from fossil CCU, but we find that this is both challenging, and not cost-efficient when accounting for residual emissions. As shown in Fig. 3 and 4 in the main text, finding a fossil CCU coordination window is difficult. This is due to the availability of CCS as an alternative cost-effective mitigation option for the steel and cement sectors, and of CDR compensation for the other sectors. A small CCU coordination window exists at an attribution of 85%,

but closes when requiring climate neutrality (compensation of residual emissions), as shown in Supplementary Fig. 9. This excludes fossil CCU CO_2 from being a viable large-scale CO_2 source for synfuels.

Supplementary Table 10**. Cost and availability of various CO_2 biogenic sources in 2050**

**Biogenic CO_2 source**	**Availability**	**Source**	**Cost**	**Source**
**Bioethanol fermentation**	0.3 $GtCO_2$ $year^{-1}$	IRENA[53]	€40.8 tCO_2^{-1}	IRENA, Rodin, Fuss[49,53,54]
**Biogas upgrading**	0.15 $GtCO_2$ $year^{-1}$	IRENA[53]	€20.1 tCO_2^{-1}	IRENA, Rodin, Fuss[49,53,54]
**Pulp and paper**	0.38 $GtCO_2$ $year^{-1}$	IRENA, Galimova[48,53]	€52.7 tCO_2^{-1}	Rodin, Fuss[49,54]
**Waste-to-energy**	0.84 $GtCO_2$ $year^{-1}$	IRENA, Galimova[48,53]	€36 tCO_2^{-1}	IRENA[53]
**Biomass for heat and power**	2.5 $GtCO_2$ $year^{-1}$	IRENA[53]	€165.6 tCO_2^{-1}	Fuss[49]

Values were calculated based on the average of the cited sources.

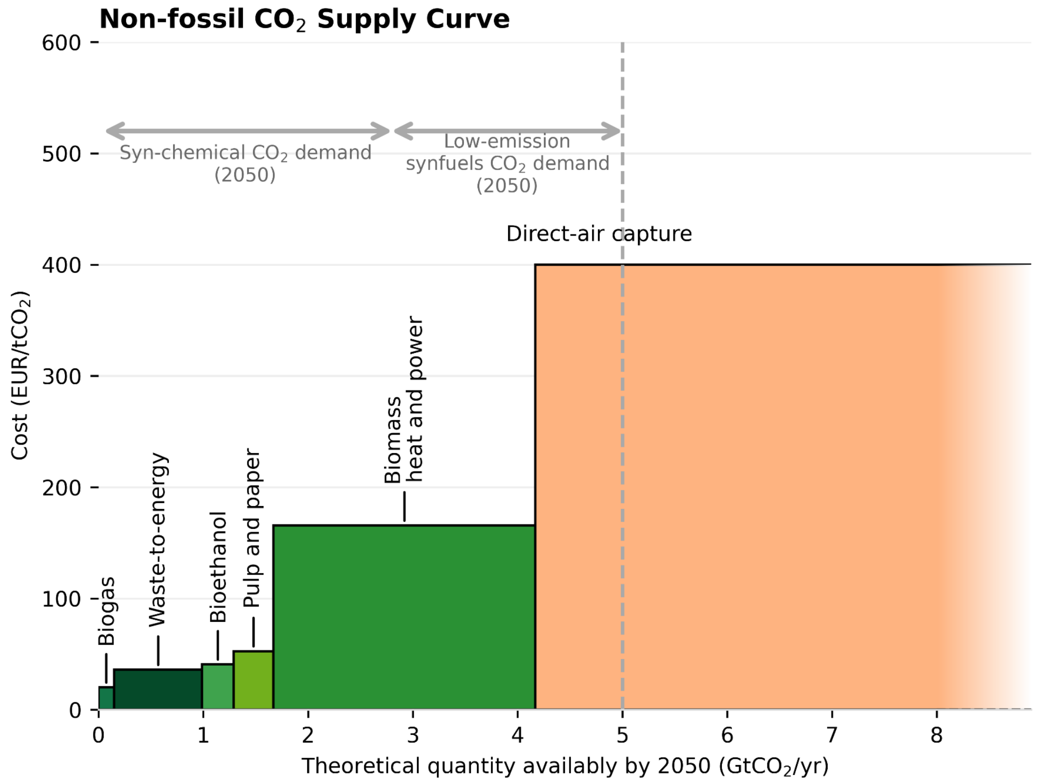

Supplementary Figure 8. **Non-fossil CO_2 supply curve for 2050.** The different biogenic CO_2 sources, with their quantity and cost, are displayed in green, and direct-air capture (atmospheric CO_2) in orange. The grey arrows indicate the expected demand for non-fossil CO_2, for low-emission synfuels in transport and synthetic methanol based-chemicals in 2050, based on Galimova et al. (2020).

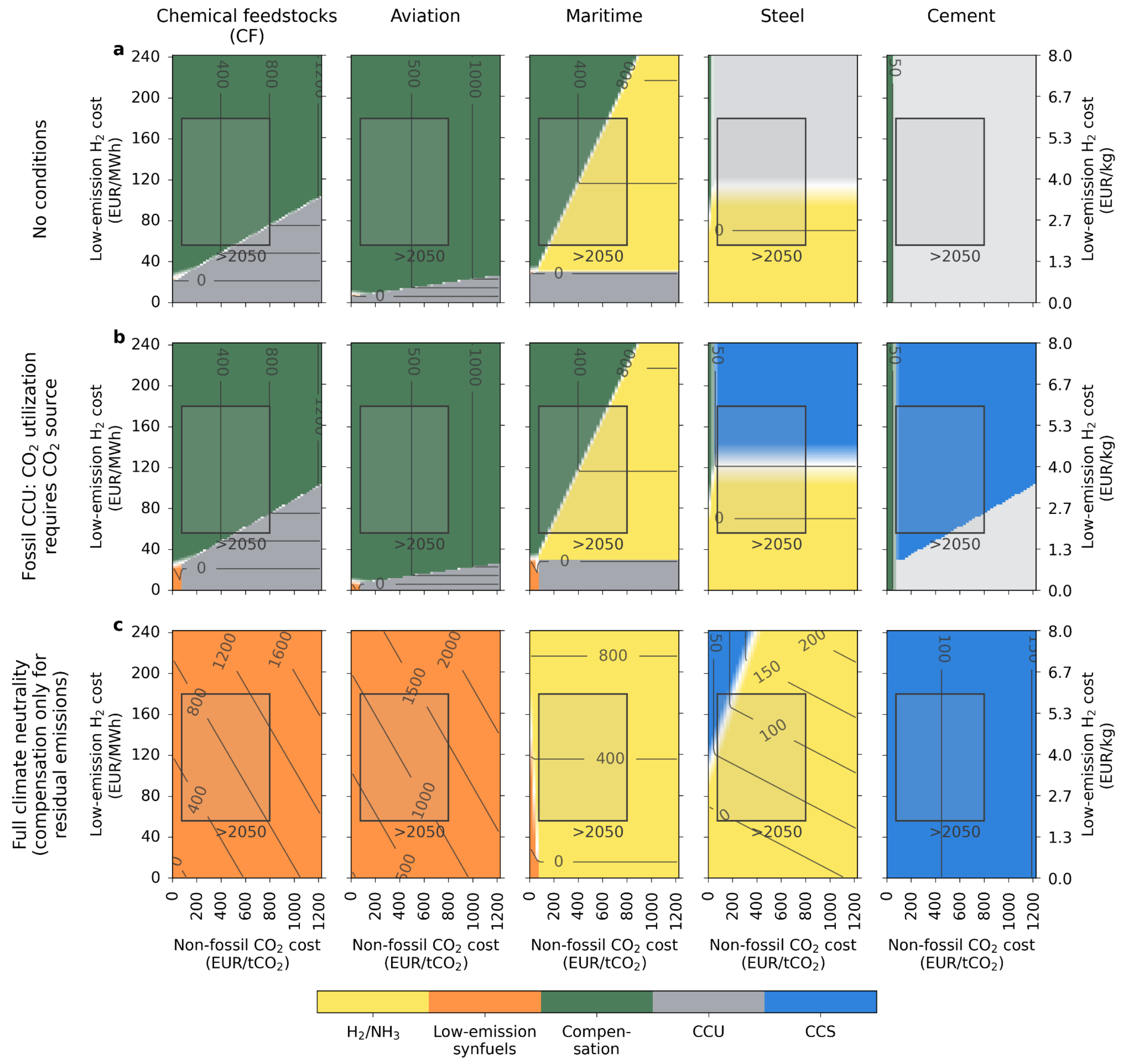

Supplementary Figure 9. **Mitigation landscapes with a higher CCU attribution of 85%.** In contrast, the base case shown in the main text uses an attribution (to the user sector) of 50%. (a) illustrates the first reference case, while (b) is a case where fossil CO_2 from CCU (carbon capture and utilization) must be sourced from the supplier sectors (steel and cement). (c) combines the previous requirement, and additionally excludes full compensation and imposes climate neutrality. H2: hydrogen, CCS: carbon capture and storage, CCU: carbon capture and utilization, NH3: ammonia

Supplementary References

1. Connelly, E., Penev, M., Elgowainy, A. & Hunter, C. *Current Status of Hydrogen Liquefaction Costs*. (2019).
2. Ghafri, S. Z. A. *et al.* Hydrogen liquefaction: a review of the fundamental physics, engineering practice and future opportunities. *Energy & Environmental Science* **15**, 2690–2731 (2022).
3. Grahn, M. *et al.* Review of electrofuel feasibility—cost and environmental impact. *Prog. Energy* **4**, 032010 (2022).
4. Stolz, B., Held, M., Georges, G. & Boulouchos, K. Techno-economic analysis of renewable fuels for ships carrying bulk cargo in Europe. *Nat Energy* **7**, 203–212 (2022).
5. Bube, S., Bullerdiek, N., Voß, S. & Kaltschmitt, M. Kerosene production from power-based syngas – A technical comparison of the Fischer-Tropsch and methanol pathway. *Fuel* **366**, 131269 (2024).
6. Energy Institute based on S&P Global Platts - Statistical Review of World Energy – with major processing by Our World in Data. 'Coal Price' [dataset]. *Our World in Data* https://ourworldindata.org/grapher/coal-prices?time=earliest..2022 (2023).
7. IPCC. *2006 IPCC Guidelines for National Greenhouse Gas Inventories*. https://www.ipcc.ch/report/2006-ipcc-guidelines-for-national-greenhouse-gas-inventories/ (2006).
8. Energy Institute based on S&P Global Platts - Statistical Review of World Energy – with major processing by Our World in Data. 'Gas Price' [dataset]. *Our World in Data* https://ourworldindata.org/grapher/natural-gas-prices (2023).
9. SB. Global 20 Ports Average Bunker Prices VLSFO. *Ship & Bunker* https://shipandbunker.com/prices/av/global/av-g20-global-20-ports-average.
10. INSEE. International prices of imported raw materials - Naphtha (European Northwest) - Spot price in euros per tonne | Insee. https://www.insee.fr/en/statistiques/serie/010002082#Tableau.
11. EIA. U.S. Kerosene-Type Jet Fuel Wholesale/Resale Price by Refiners (Dollars per Gallon). https://www.eia.gov/dnav/pet/hist/LeafHandler.ashx?n=pet&s=ema_epjk_pwg_nus_dpg&f=m

(2022).

12. Mukhopadhaya, J. & Rutherford, D. *Performance Analysis of Evolutionary Hydrogen-Powered Aircraft*. (2022).
13. IEAGHG. *Iron and Steel CCS Study (Techno-Economics Integrated Steel Mill)*. https://ieaghg.org/docs/General_Docs/Reports/2013-04.pdf (2013).
14. Al-Shamma, O. A Comparative Study of Cost Estimation Models used For Preliminary Aircraft Design. *Global Journals of Research in Engineering* **14**, 9–18 (2014).
15. Dahal, K. *et al.* Techno-economic review of alternative fuels and propulsion systems for the aviation sector. *Renewable and Sustainable Energy Reviews* **151**, 111564 (2021).
16. IEA. ETP Clean Energy Technology Guide. https://www.iea.org/data-and-statistics/data-tools/etp-clean-energy-technology-guide (2023).
17. Korberg, A. D., Brynolf, S., Grahn, M. & Skov, I. R. Techno-economic assessment of advanced fuels and propulsion systems in future fossil-free ships. *Renewable and Sustainable Energy Reviews* **142**, 110861 (2021).
18. Anwar, S., Zia, M. Y. I., Rashid, M., Rubens, G. Z. de & Enevoldsen, P. Towards Ferry Electrification in the Maritime Sector. *Energies* **13**, 6506 (2020).
19. McKinlay, C. J., Turnock, S. R. & Hudson, D. A. Route to zero emission shipping: Hydrogen, ammonia or methanol? *International Journal of Hydrogen Energy* **46**, 28282–28297 (2021).
20. Ustolin, F., Campari, A. & Taccani, R. An Extensive Review of Liquid Hydrogen in Transportation with Focus on the Maritime Sector. *Journal of Marine Science and Engineering* **10**, 1222 (2022).
21. Gray, N., McDonagh, S., O'Shea, R., Smyth, B. & Murphy, J. D. Decarbonising ships, planes and trucks: An analysis of suitable low-carbon fuels for the maritime, aviation and haulage sectors. *Advances in Applied Energy* **1**, 100008 (2021).
22. Bertagni, M. B. *et al.* Minimizing the impacts of the ammonia economy on the nitrogen cycle and climate. *Proceedings of the National Academy of Sciences* **120**, e2311728120 (2023).
23. Wolfram, P., Kyle, P., Zhang, X., Gkantonas, S. & Smith, S. Using ammonia as a shipping fuel could disturb the nitrogen cycle. *Nat Energy* **7**, 1112–1114 (2022).

24. Green Shipping Programme. *Ammonia-Powered Bulkcarrier. Pilot Report*. https://greenshippingprogramme.com/wp-content/uploads/2023/06/Ammonia-powered-bulk-carrier.-Pilot-report..pdf (2023).
25. Maersk Plans First Methanol Retrofit for In-Service Containership. *The Maritime Executive* https://maritime-executive.com/article/maersk-plans-first-methanol-retrofit-for-in-service-containership (2021).
26. COSCO Signs Up for Methanol Retrofits. *MAN Energy Solutions* https://www.man-es.com/company/press-releases/press-details/2023/12/07/cosco-signs-up-for-methanol-retrofits (2023).
27. Inal, O. B., Zincir, B. & Deniz, C. Investigation on the decarbonization of shipping: An approach to hydrogen and ammonia. *International Journal of Hydrogen Energy* **47**, 19888–19900 (2022).
28. de Vries, N. Safe and effective application of ammonia as a marine fuel. (2019).
29. Fischedick, M., Marzinkowski, J., Winzer, P. & Weigel, M. Techno-economic evaluation of innovative steel production technologies. *Journal of Cleaner Production* **84**, 563–580 (2014).
30. Agora Energiewende, FutureCamp, Wuppertal Institut und Ecologic Institut. *Klimaschutzverträge für die Industrie- transformation. Aktualisierte Analyse zur Stahlbranche.* 88 (2021).
31. Vogl, V., Åhman, M. & Nilsson, L. J. Assessment of hydrogen direct reduction for fossil-free steelmaking. *Journal of Cleaner Production* **203**, 736–745 (2018).
32. Jacobasch, E. *et al.* Economic evaluation of low-carbon steelmaking via coupling of electrolysis and direct reduction. *Journal of Cleaner Production* **328**, 129502 (2021).
33. De Lena, E. *et al.* Techno-economic analysis of calcium looping processes for low CO2 emission cement plants. *International Journal of Greenhouse Gas Control* **82**, 244–260 (2019).
34. Voldsund, M. *et al.* Comparison of Technologies for CO2 Capture from Cement Production—Part 1: Technical Evaluation. *Energies* **12**, 559 (2019).
35. Gardarsdottir, S. O. *et al.* Comparison of Technologies for CO2 Capture from Cement Production—Part 2: Cost Analysis. *Energies* **12**, 542 (2019).
36. Levi, P. G. & Cullen, J. M. Mapping Global Flows of Chemicals: From Fossil Fuel Feedstocks to Chemical Products. *Environ. Sci. Technol.* **52**, 1725–1734 (2018).

37. Kätelhön, A., Meys, R., Deutz, S., Suh, S. & Bardow, A. Climate change mitigation potential of carbon capture and utilization in the chemical industry. *PNAS* **116**, 11187–11194 (2019).

38. Lopez, G. A. A., Keiner, D., Fasihi, M., Koiranen, T. & Breyer, C. From fossil to green chemicals: Sustainable pathways and new carbon feedstocks for the global chemical industry. *Energy Environ. Sci.* **16**, 2879–2909 (2023).

39. Spallina, V. *et al.* Techno-economic assessment of different routes for olefins production through the oxidative coupling of methane (OCM): Advances in benchmark technologies. *Energy Conversion and Management* **154**, 244–261 (2017).

40. Dutta, A., Karimi, I. A. & Farooq, S. Technoeconomic Perspective on Natural Gas Liquids and Methanol as Potential Feedstocks for Producing Olefins. *Ind. Eng. Chem. Res.* **58**, 963–972 (2019).

41. Ren, T., Patel, M. & Blok, K. Olefins from conventional and heavy feedstocks: Energy use in steam cracking and alternative processes. *Energy* **31**, 425–451 (2006).

42. Geyer, R., Jambeck, J. R. & Law, K. L. Production, use, and fate of all plastics ever made. *Science Advances* **3**, e1700782 (2017).

43. George, J. F., Müller, V. P., Winkler, J. & Ragwitz, M. Is blue hydrogen a bridging technology? - The limits of a CO2 price and the role of state-induced price components for green hydrogen production in Germany. *Energy Policy* **167**, 113072 (2022).

44. Howarth, R. W. & Jacobson, M. Z. How green is blue hydrogen? *Energy Science & Engineering* **9**, 1676–1687 (2021).

45. Rosenow, J. & Lowes, R. Will blue hydrogen lock us into fossil fuels forever? *One Earth* **4**, 1527–1529 (2021).

46. IEAGHG. *Techno - Economic Evaluation of SMR Based Standalone (Merchant) Hydrogen Plant with CCS*. (2017).

47. Lewis, E. *et al. Comparison of Commercial, State-of-the-Art, Fossil-Based Hydrogen Production Technologies*. https://www.osti.gov/biblio/1862910 (2022) doi:10.2172/1862910.

48. Galimova, T. *et al.* Global demand analysis for carbon dioxide as raw material from key industrial sources and direct air capture to produce renewable electricity-based fuels and

chemicals. *Journal of Cleaner Production* **373**, 133920 (2022).

49. Fuss, S. *et al.* Negative emissions—Part 2: Costs, potentials and side effects. *Environ. Res. Lett.* **13**, 063002 (2018).

50. McKenna, B., Poehler, A., Quin, K., Lam, Y. & Tempke, R. *Global Availability of Biogenic CO2 and Implications for Maritime Decarbonization*. (2024).

51. Creutzig, F. *et al.* Bioenergy and climate change mitigation: an assessment. *GCB Bioenergy* **7**, 916–944 (2015).

52. Transport and Environment. *EUROPEAN CO2 AVAILABILITY FROM POINT-SOURCES AND DIRECT AIR CAPTURE*. https://www.transportenvironment.org/articles/scaling-up-direct-air-capture (2022).

53. IRENA AND METHANOL INSTITUTE. *Innovation Outlook : Renewable Methanol*. (2021).

54. Rodin, V., Lindorfer, J., Böhm, H. & Vieira, L. Assessing the potential of carbon dioxide valorisation in Europe with focus on biogenic CO2. *Journal of CO2 Utilization* **41**, 101219 (2020).
